# Supplementary material for: A set of Arabidopsis genes involved in the accommodation of the downy mildew pathogen Hyaloperonospora arabidopsidis
Source: PLoS Pathog. 2019 Jul 12;15(7):e1007747. doi: 10.1371/journal.ppat.1007747 (PMC6625732; doi:10.1371/journal.ppat.1007747)
Supplement: S1 Fig — TAIR/GenBank protein identifiers are shown. Red, consensus level high = 90%; blue, consensus level low = 50%. Alignments were generated using the multiple sequence alignment software by Corpet (Corpet, 1988). (DOCX) [file ppat.1007747.s001.docx]

**
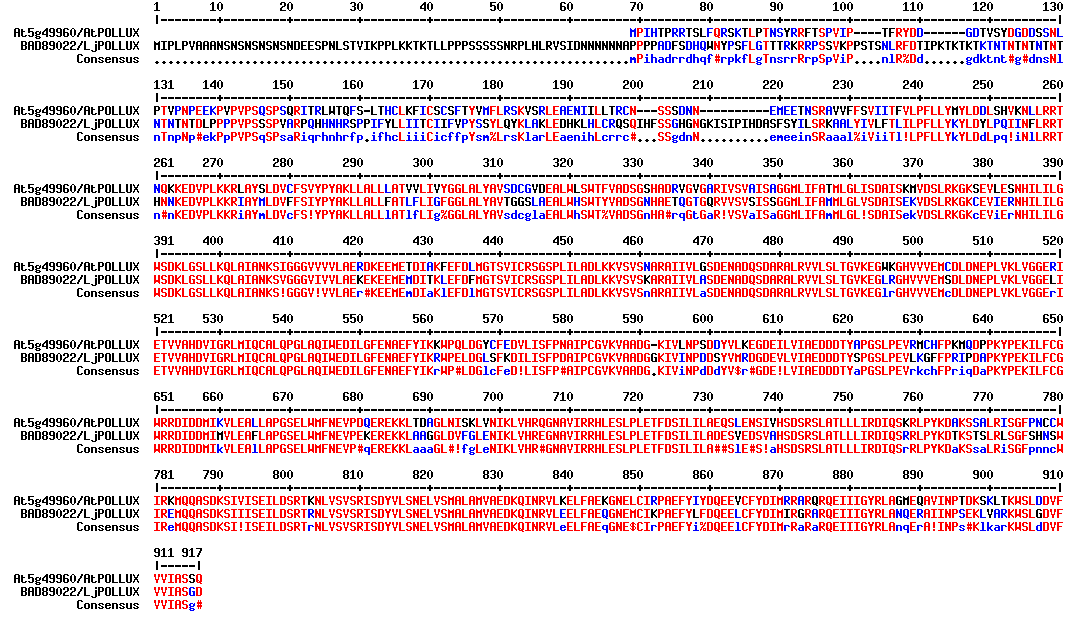
**

**---------------------------------------------------------------------------------------------------------------------**

**
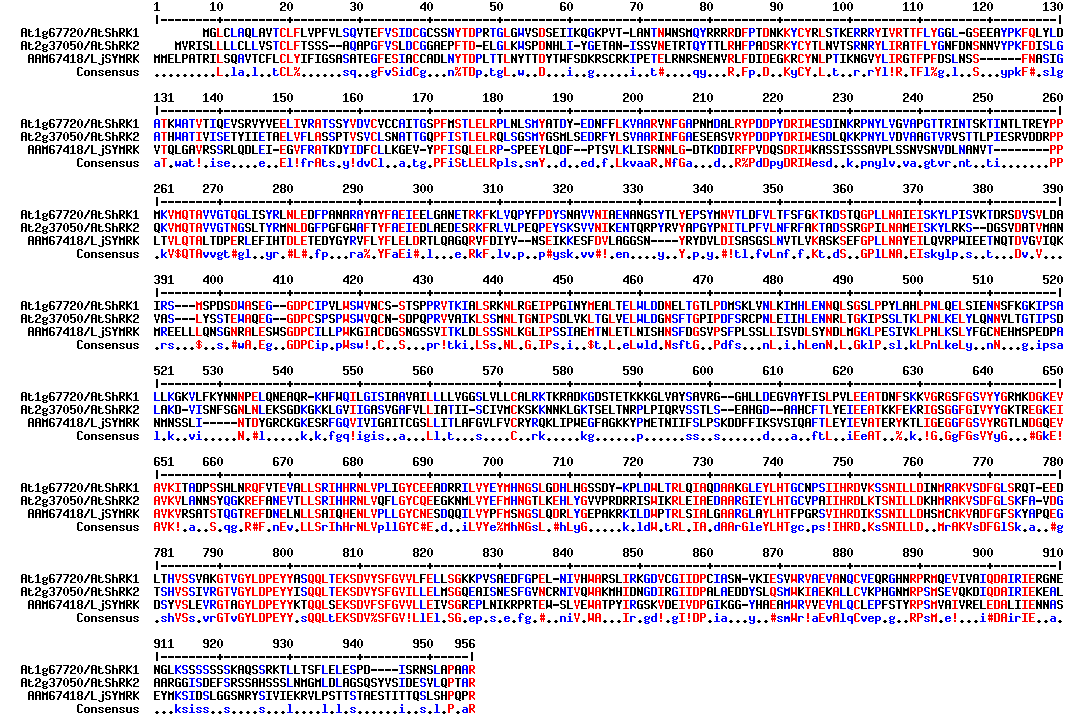
**

**--------------------------------------------------------------------------------------------------------------------**

**
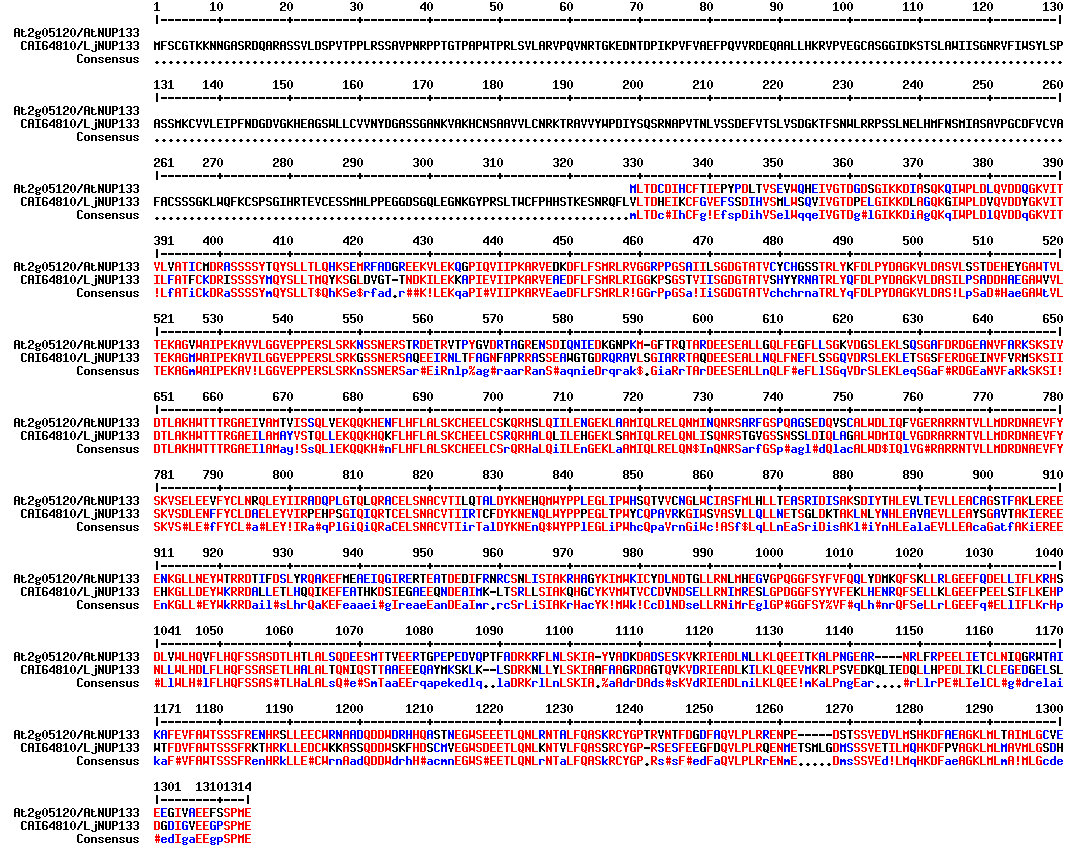
**

**---------------------------------------------------------------------------------------------------------------------**

**
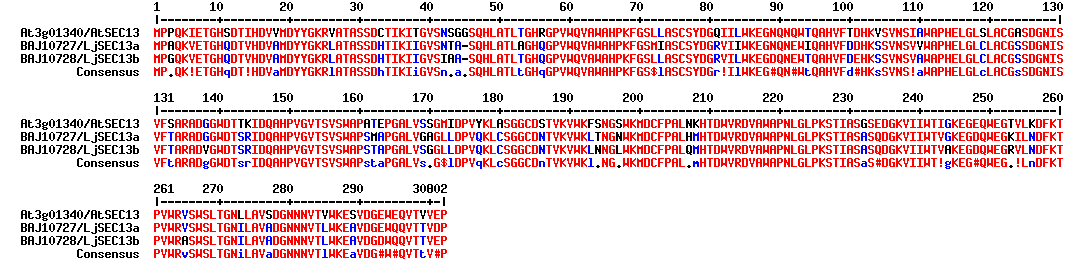
**S1 Fig. Alignments of the protein sequences of *L. japonicus* CSGs and closely related *A. thaliana* SNUPO genes.

TAIR/GenBank protein identifiers are shown. Red, consensus level high = 90%; blue, consensus level low = 50%. Alignments were generated using the multiple sequence alignment software by Corpet (Corpet, 1988).
