# Supplementary material for: A set of Arabidopsis genes involved in the accommodation of the downy mildew pathogen Hyaloperonospora arabidopsidis
Source: PLoS Pathog. 2019 Jul 12;15(7):e1007747. doi: 10.1371/journal.ppat.1007747 (PMC6625732; doi:10.1371/journal.ppat.1007747)
Supplement: S2 Fig — TAIR/GenBank protein identifiers are shown; dashed lines, positions of introns in the original gene sequence; red triangles, positions of T-DNA insertion in the respective mutants; SP, signal peptide; MLD, malectin-like domain; LRRs, leucine-rich repeats; TM, transmembrane domain; KD, kinase domain. (DOCX) [file ppat.1007747.s002.docx]

**
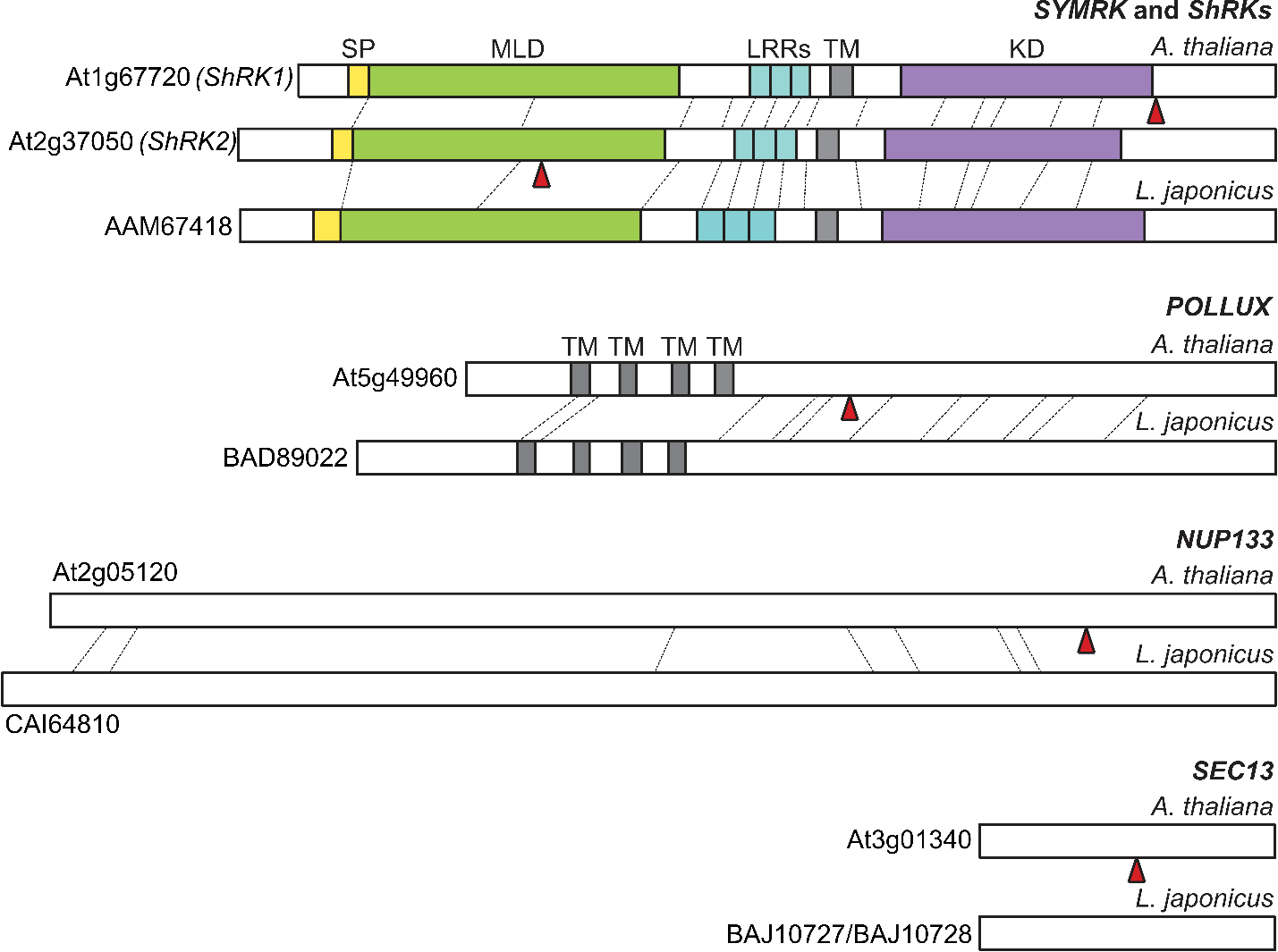
**

# S2 Fig. Comparison of gene structures and protein domains of *L. japonicus* CSGs and closely related *A. thaliana* SNUPO genes*.*

TAIR/GenBank protein identifiers are shown; dashed lines, positions of introns in the original gene sequence; red triangles, positions of T-DNA insertion in the respective mutants; SP, signal peptide; MLD, malectin-like domain; LRRs, leucine-rich repeats; TM, transmembrane domain; KD, kinase domain.
