# Supplementary material for: A set of Arabidopsis genes involved in the accommodation of the downy mildew pathogen Hyaloperonospora arabidopsidis
Source: PLoS Pathog. 2019 Jul 12;15(7):e1007747. doi: 10.1371/journal.ppat.1007747 (PMC6625732; doi:10.1371/journal.ppat.1007747)
Supplement: S3 Fig — The specific domain composition featuring a MLD followed by the conserved gly-asp-pro-cys (GDPC) motif known to be required for the proper establishment of the symbiotic program in the root epidermis (Kosuta et al., 2011; Antolín-Llovera et al., 2014), and LRRs is not restricted to SYMRK but can also be found in 42 of the 50 members of LRR I-RLKs present in A. thaliana (Shiu & Bleecker, 2003; Hok et al., 2011). A maximum likelihood phylogenetic tree based on the extracytoplasmic regions (excluding the signal peptides) of 42 MLD-LRR-RK proteins from A. thaliana and SYMRK from L. japonicus. Numbers on each node represent the respective bootstrap values. Bar, relative genetic distance (arbitrary unit). (DOCX) [file ppat.1007747.s003.docx]

**
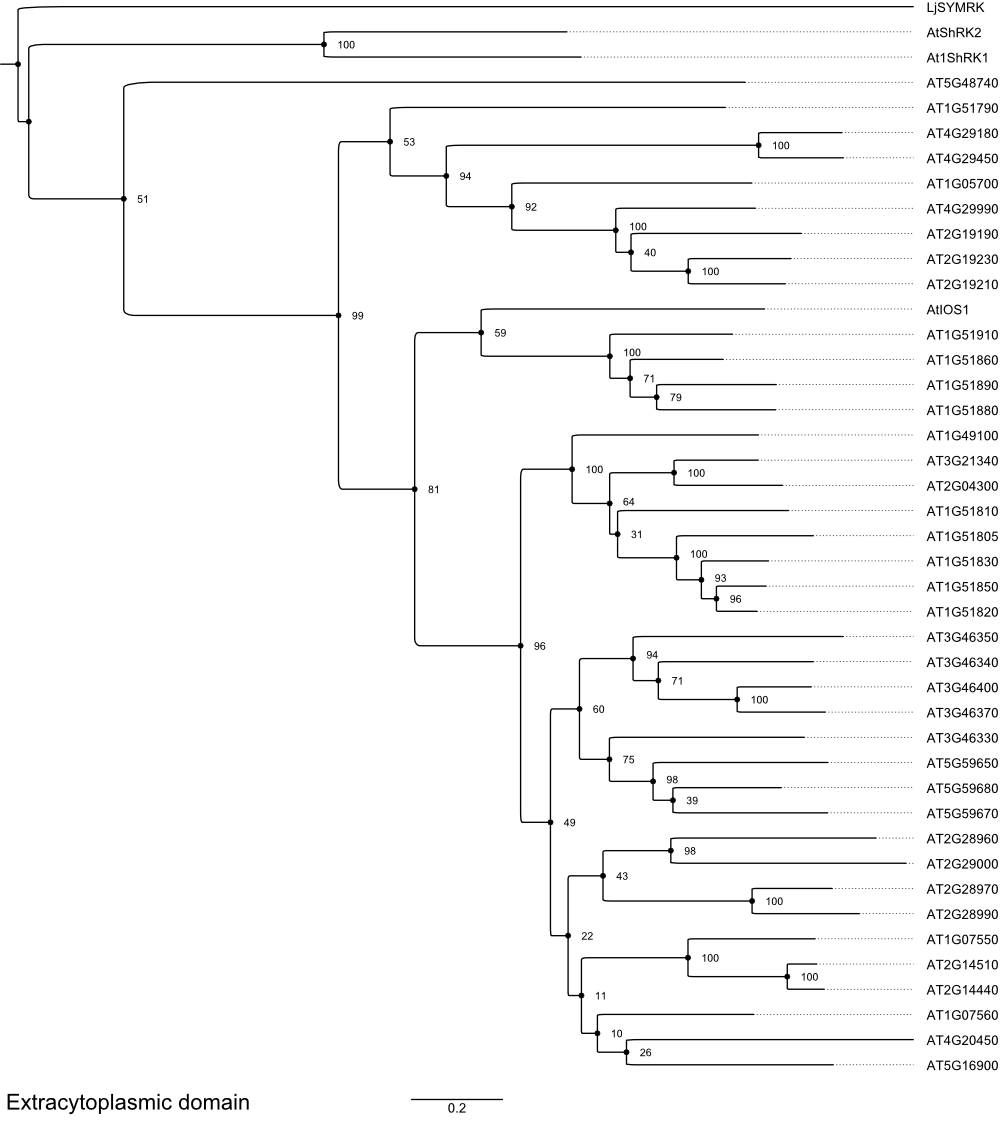
**

**S3 Fig. Maximum likelihood phylogenetic tree of the extracytoplasmic domains of MLD-LRR-RKs from *A. thaliana* and *L. japonicus* SYMRK.** The specific domain composition featuring a MLD followed by the conserved gly-asp-pro-cys (GDPC) motif known to be required for the proper establishment of the symbiotic program in the root epidermis (Kosuta *et al.*, 2011; Antolín-Llovera *et al.*, 2014), and LRRs is not restricted to SYMRK but can also be found in 42 of the 50 members of LRR I-RLKs present in *A. thaliana* (Shiu & Bleecker, 2003; Hok *et al.*, 2011). A maximum likelihood phylogenetic tree based on the extracytoplasmic regions (excluding the signal peptides) of 42 MLD-LRR-RK proteins from *A. thaliana* and SYMRK from *L. japonicus*. Numbers on each node represent the respective bootstrap values. Bar, relative genetic distance (arbitrary unit).
