# Supplementary material for: A set of Arabidopsis genes involved in the accommodation of the downy mildew pathogen Hyaloperonospora arabidopsidis
Source: PLoS Pathog. 2019 Jul 12;15(7):e1007747. doi: 10.1371/journal.ppat.1007747 (PMC6625732; doi:10.1371/journal.ppat.1007747)
Supplement: S4 Fig — A maximum likelihood phylogenetic tree based on the highly conserved kinase domains of 42 MLD-LRR-RK proteins from A. thaliana and SYMRK from L. japonicus. Numbers on each node represent the respective bootstrap values. Bar, relative genetic distance (arbitrary unit). (DOCX) [file ppat.1007747.s004.docx]

**
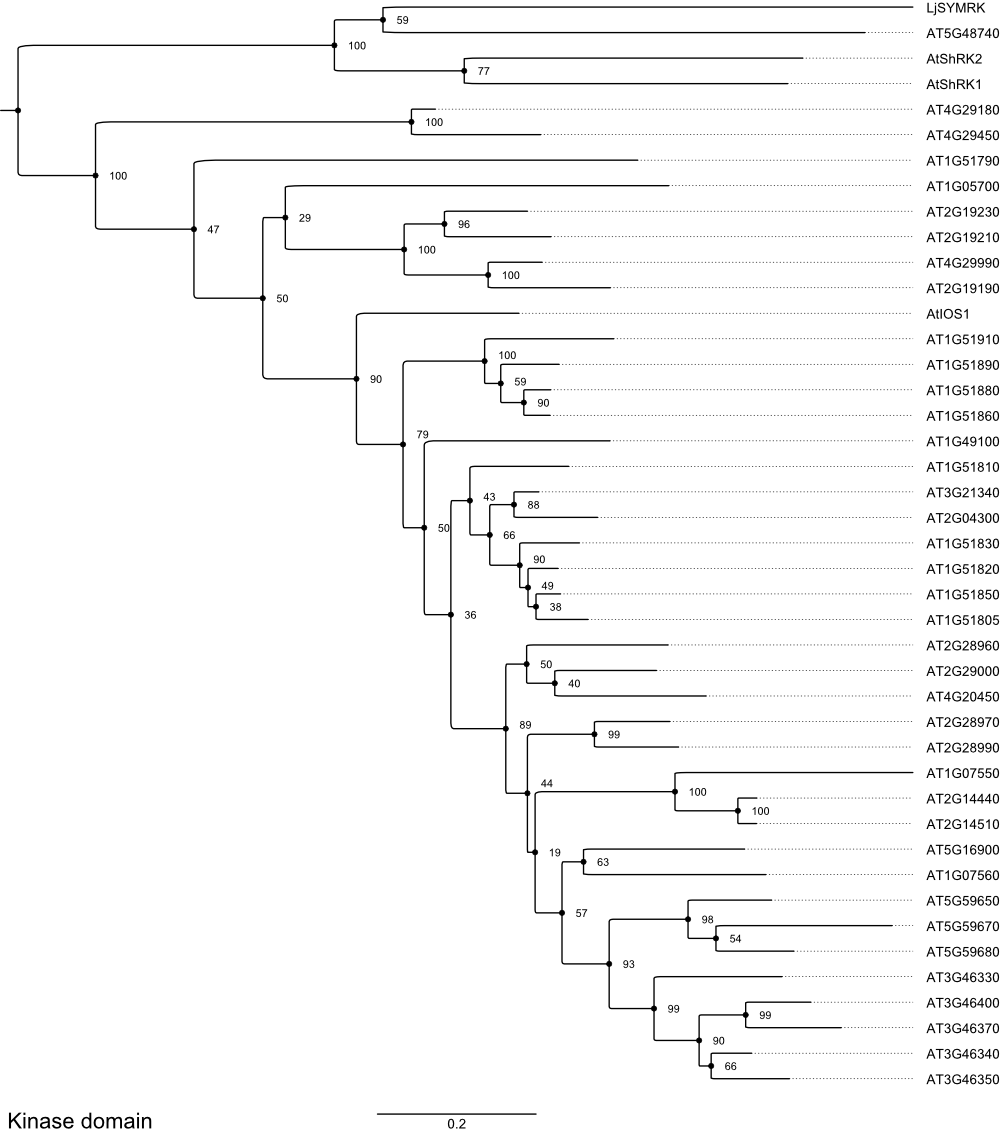
**

**S4 Fig. Maximum likelihood phylogenetic tree of the highly conserved kinase domains of MLD-LRR-RKs from *A. thaliana* and *L. japonicus* SYMRK.**

A maximum likelihood phylogenetic tree based on the highly conserved kinase domains of 42 MLD-LRR-RK proteins from *A. thaliana* and SYMRK from *L. japonicus*. Numbers on each node represent the respective bootstrap values. Bar, relative genetic distance (arbitrary unit).
