# Supplementary material for: A set of Arabidopsis genes involved in the accommodation of the downy mildew pathogen Hyaloperonospora arabidopsidis
Source: PLoS Pathog. 2019 Jul 12;15(7):e1007747. doi: 10.1371/journal.ppat.1007747 (PMC6625732; doi:10.1371/journal.ppat.1007747)
Supplement: S5 Fig — Bar charts represent the mean number of sporangiophores ± s.e.m on infected cotyledons of A. thaliana wild-type (Col-0) or the indicated mutants 4 dpi with Hpa isolate Noco2 in two additional replicates (a + b). n = 21–99. Stars indicate significant differences to Col-0 (Wilcoxon–Mann–Whitney test with Bonferroni-Holm correction; *, p < 0.05; **, p < 0.01; ***, p < 0.001). (DOCX) [file ppat.1007747.s005.docx]

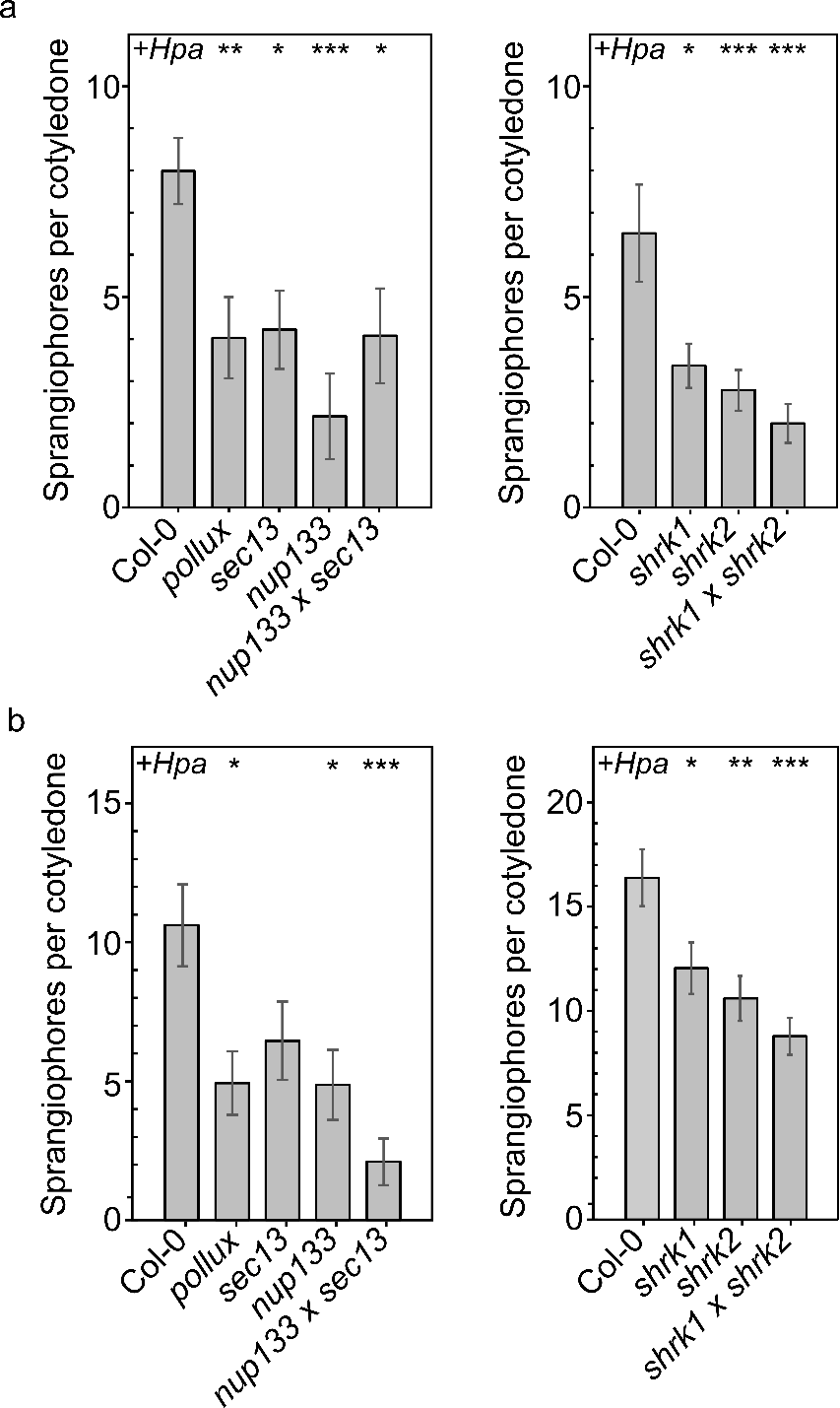


**S5 Fig.** **Mutations in *A. thaliana* SNUPO genes reduce the reproductive success of the oomycete downy mildew pathogen *H. arabidopsidis.***

Bar charts represent the mean number of sporangiophores ± s.e.m on infected cotyledons of *A. thaliana* wild-type (Col-0) or the indicated mutants 4 dpi with *Hpa* isolate NoCo2 in two additional replicates **(a + b)**. n = 21 – 99. Stars indicate significant differences to Col-0 (Wilcoxon–Mann–Whitney test with Bonferroni-Holm correction; *, p < 0.05; **, p < 0.01; ***, p < 0.001).
