# Supplementary material for: A set of Arabidopsis genes involved in the accommodation of the downy mildew pathogen Hyaloperonospora arabidopsidis
Source: PLoS Pathog. 2019 Jul 12;15(7):e1007747. doi: 10.1371/journal.ppat.1007747 (PMC6625732; doi:10.1371/journal.ppat.1007747)
Supplement: S6 Fig — Boxplots represent the percentage of haustoria-containing cells per cells contacted by hyphae on leaves of A. thaliana wild-type (Col-0), the indicated mutants and the transgenic complementation lines (pollux co, pPOLLUX:POLLUX) 5 dpi with Hpa isolate Noco2. For each genotype, at least ten independent stretches of hyphae per leaf have been analysed on at least 5 leaves. Black circles, data points outside 1.5 IQR of the upper/lower quartile; bold black line, median; box, IQR; whiskers, lowest/highest data point within 1.5 IQR of the lower/upper quartile. Stars indicate significant differences to Col-0 (Wilcoxon–Mann–Whitney test with Bonferroni-Holm correction; *, p < 0.05). (DOCX) [file ppat.1007747.s006.docx]

**
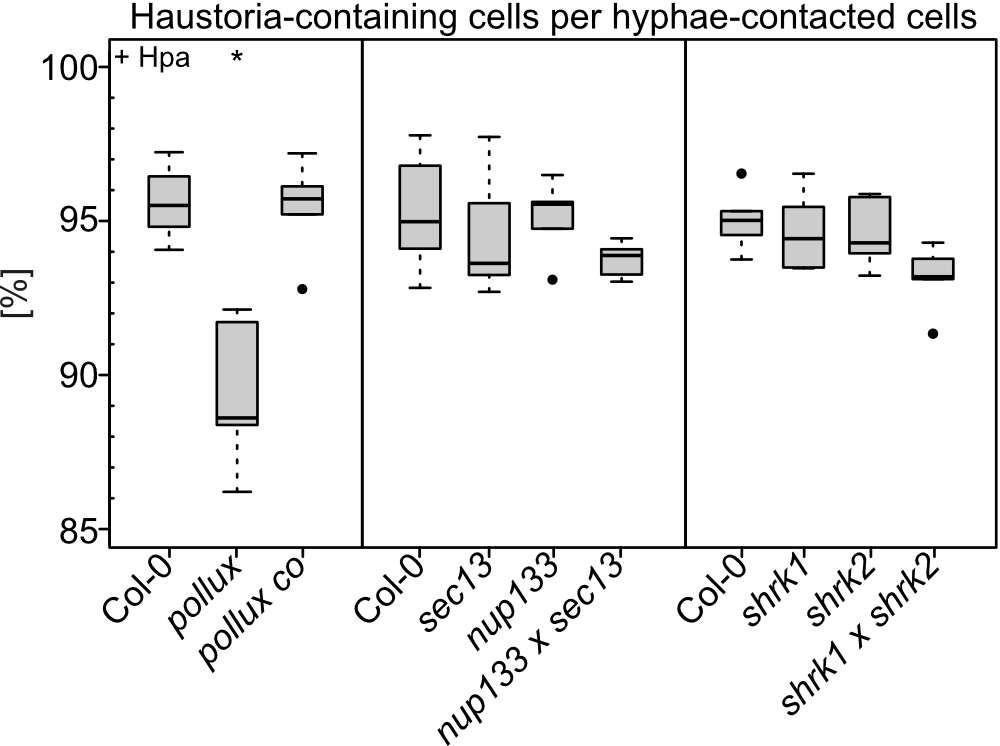
**

**S6 Fig. Mutations in *A. thaliana* SNUPO genes do not reduce the number of cells that accommodate haustoria*.***

Boxplots represent the percentage of haustoria-containing cells per cells contacted by hyphae on leaves of *A. thaliana* wild-type (Col-0), the indicated mutants and the transgenic complementation lines (*pollux co, pPOLLUX:POLLUX*) 5 dpi with *Hpa* isolate NoCo2. For each genotype, at least ten independent stretches of hyphae per leaf have been analysed on at least 5 leaves. Black circles, data points outside 1.5 IQR of the upper/lower quartile; bold black line, median; box, IQR; whiskers, lowest/highest data point within 1.5 IQR of the lower/upper quartile. Stars indicate significant differences to Col-0 (Wilcoxon–Mann–Whitney test with Bonferroni-Holm correction; *, p < 0.05).
