# Supplementary material for: A set of Arabidopsis genes involved in the accommodation of the downy mildew pathogen Hyaloperonospora arabidopsidis
Source: PLoS Pathog. 2019 Jul 12;15(7):e1007747. doi: 10.1371/journal.ppat.1007747 (PMC6625732; doi:10.1371/journal.ppat.1007747)
Supplement: S8 Fig — Boxplots represent the percentage of multilobed haustoria among total haustoria on A. thaliana wild-type (Col-0; Ler), and ios1 mutant lines 5 dpi with Hpa isolate Noco2 (a), 5 dpi with Hpa isolate Noco2 and Waco9. For each genotype, at least ten independent stretches of hyphae per leaf have been analysed on at least 5 leaves. Black circles, data points outside 1.5 IQR of the upper/lower quartile; bold black line, median; box, IQR; whiskers, lowest/highest data point within 1.5 IQR of the lower/upper quartile. Stars indicate significant differences to Col-0 (Wilcoxon–Mann–Whitney test with Bonferroni-Holm correction; *, p < 0.05). (DOCX) [file ppat.1007747.s008.docx]

**
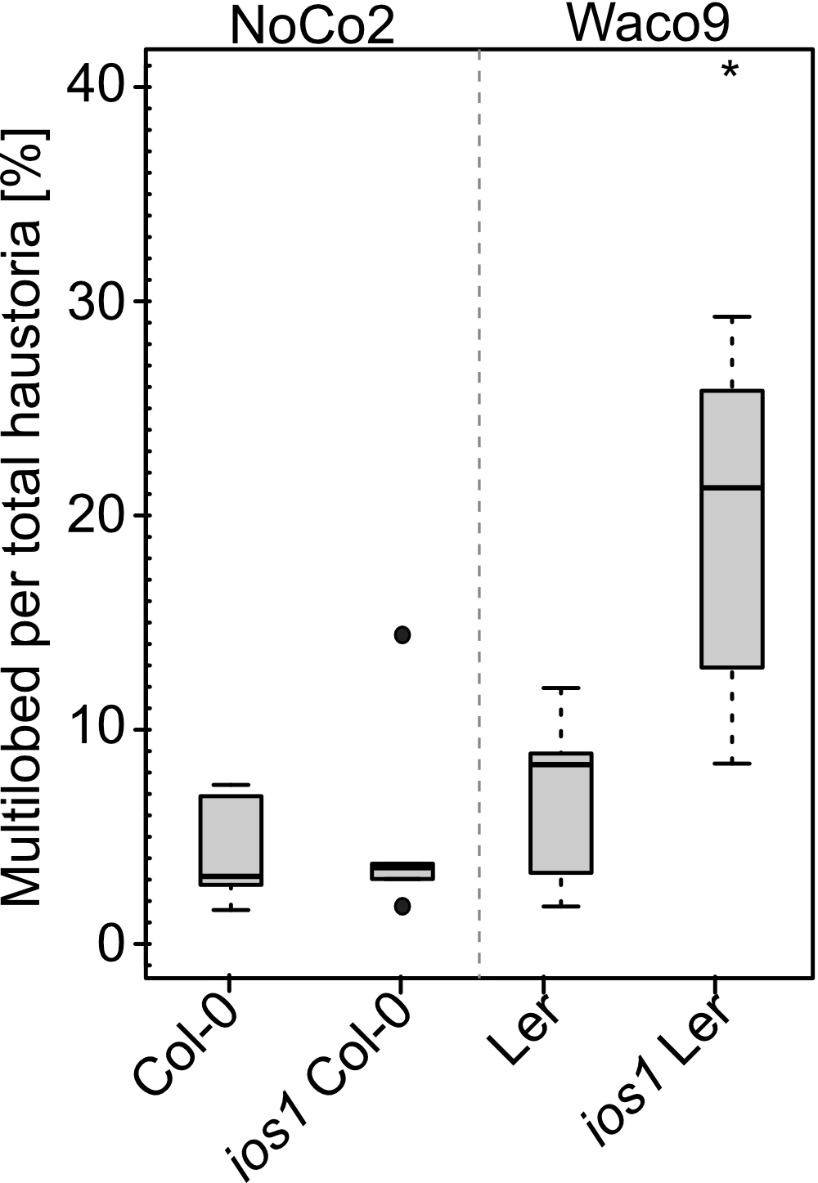
**

**S8 Fig.** **An increased frequency of multilobed *Hpa* haustoria in a *A. thaliana ios1* mutant was observed in the Ler background infected with Waco9, but not in the Col-0 background infected with NoCo2.**

Boxplots represent the percentage of multilobed haustoria among total haustoria on *A. thaliana* wild-type (Col-0; Ler), and *ios1* mutant lines 5 dpi with *Hpa* isolate NoCo2 (a), 5 dpi with *Hpa* isolate NoCo2 and Waco9. For each genotype, at least ten independent stretches of hyphae per leaf have been analysed on at least 5 leaves. Black circles, data points outside 1.5 IQR of the upper/lower quartile; bold black line, median; box, IQR; whiskers, lowest/highest data point within 1.5 IQR of the lower/upper quartile. Stars indicate significant differences to Col-0 (Wilcoxon–Mann–Whitney test with Bonferroni-Holm correction; *, p < 0.05).
