# Supplementary material for: A set of Arabidopsis genes involved in the accommodation of the downy mildew pathogen Hyaloperonospora arabidopsidis
Source: PLoS Pathog. 2019 Jul 12;15(7):e1007747. doi: 10.1371/journal.ppat.1007747 (PMC6625732; doi:10.1371/journal.ppat.1007747)
Supplement: S9 Fig — Bar charts represent the mean number of conidiophores ± s.e.m on leaves of the indicated mutants relative to the wild-type (Col-0) 5 dpi with E. cruciferarum. At least ten colonies per leaf on at least 6 leaves have been counted. Results originate from an independent biological repetition of the experiment shown in Fig 3. No significant differences to Col-0 were detected. (DOCX) [file ppat.1007747.s009.docx]

**
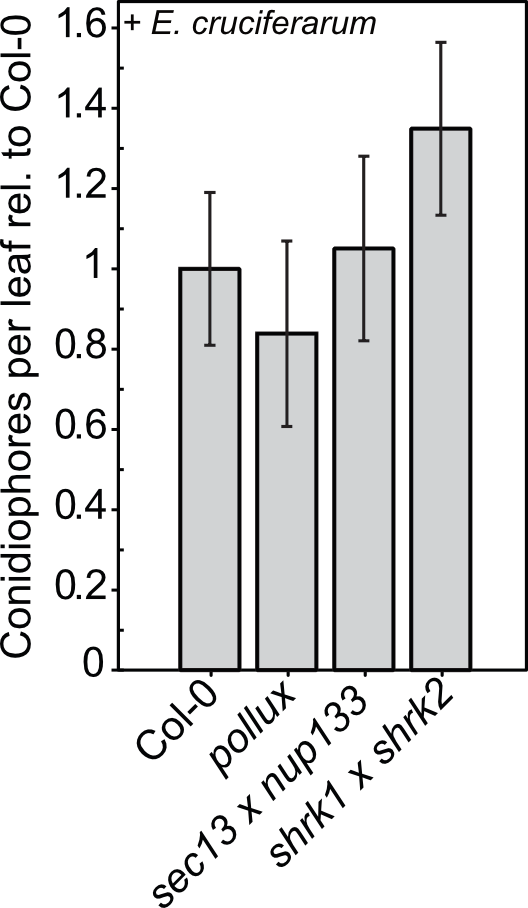
**

**S9 Fig.**  **Mutations in *A. thaliana* SNUPO genes do not impair the reproductive success of the fungal powdery mildew pathogen *E. cruciferarum*.**

Bar charts represent the mean number of conidiophores ± s.e.m on leaves of the indicated mutants relative to the wild-type (Col-0) 5 dpi with *E. cruciferarum*. At least ten colonies per leaf on at least 6 leaves have been counted. No significant differences to Col-0 were detected.
