# Supplementary material for: A set of Arabidopsis genes involved in the accommodation of the downy mildew pathogen Hyaloperonospora arabidopsidis
Source: PLoS Pathog. 2019 Jul 12;15(7):e1007747. doi: 10.1371/journal.ppat.1007747 (PMC6625732; doi:10.1371/journal.ppat.1007747)
Supplement: S10 Fig — 3-week-old A. thaliana wild-type (Col-0) plants grown alongside the indicated mutant lines under long day conditions. The dwarf phenotype of snc1 is included on the far right for comparison. (DOCX) [file ppat.1007747.s010.docx]

**
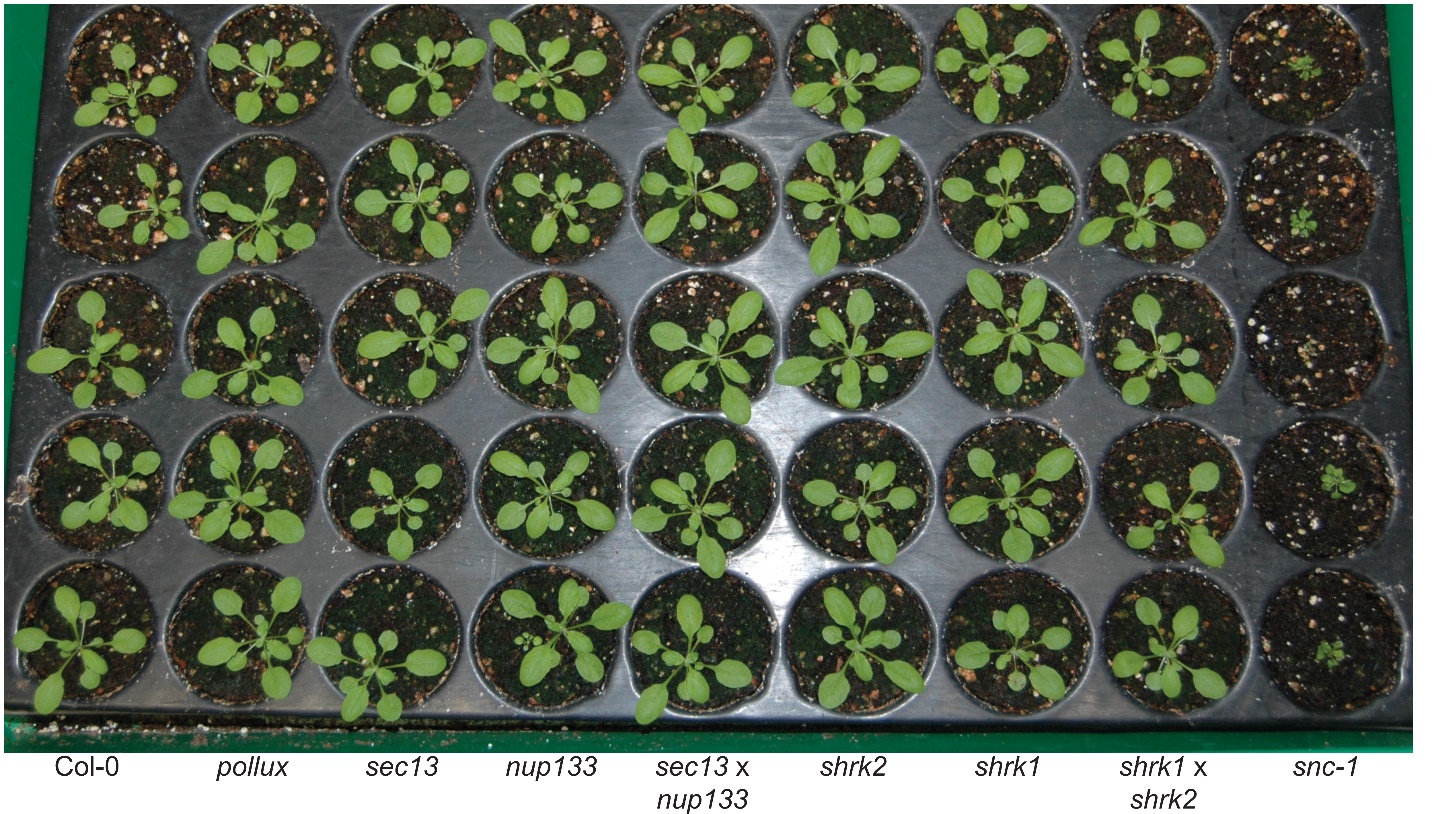
**

**S10 Fig. *A. thaliana* SNUPO mutants do not show developmental or growth defects.**

3-week-old *A. thaliana* wild-type (Col-0) plants grown alongside the indicated mutant lines under long day conditions. The dwarf phenotype of *snc1* is included on the far right for comparison.
