# Supplementary material for: A set of Arabidopsis genes involved in the accommodation of the downy mildew pathogen Hyaloperonospora arabidopsidis
Source: PLoS Pathog. 2019 Jul 12;15(7):e1007747. doi: 10.1371/journal.ppat.1007747 (PMC6625732; doi:10.1371/journal.ppat.1007747)
Supplement: S1 Table — Sequence identifiers and identities / similarities shared between the protein sequences of A. thaliana SNUPO and other nucleoporin genes and those of their respective L. japonicus counterparts. The numbers for AtSEC13 indicate the identity / similarity of its amino acid sequence to each of the two predicted LjSEC13 proteins. For ShRK1 and ShRK2, numbers in brackets refer to their kinase domain (KD) only. n.d., not detected. NASC ID: insertion mutant identifier. (DOCX) [file ppat.1007747.s011.docx]

**S1** **Table *A. thaliana* SNUPO genes with encoded proteins and their respective identities / similarities to related *L. japonicus* genes.**

Sequence identifiers and identities / similarities shared between the protein sequences of *A. thaliana* SNUPO and other nucleoporin genes and those of their respective *L. japonicus* counterparts. The numbers for AtSEC13 indicate the identity / similarity of its amino acid sequence to each of the two predicted LjSEC13 proteins. For ShRK1 and ShRK2, numbers in brackets refer to their kinase domain (KD) only. n.d., not detected. NASC ID: insertion mutant identifier.
