# Supplementary material for: A set of Arabidopsis genes involved in the accommodation of the downy mildew pathogen Hyaloperonospora arabidopsidis
Source: PLoS Pathog. 2019 Jul 12;15(7):e1007747. doi: 10.1371/journal.ppat.1007747 (PMC6625732; doi:10.1371/journal.ppat.1007747)
Supplement: S2 Table — Constructs labelled with “GG” were generated via Golden Gate cloning. For details on assembly method, general modules and plasmids (Gxx, BBxx), see (Binder et al., 2014). Golden Gate constructs contain silent mutations to facilitate cloning. (DOCX) [file ppat.1007747.s012.docx]

**S2** **Table Constructs and cloning strategy.**

Constructs labelled with “GG” were generated via Golden Gate cloning. For details on assembly method, general modules and plasmids (Gxx, BBxx), see (Binder *et al.*, 2014). Golden Gate constructs contain silent mutations to facilitate cloning.

| **Entry clones / Golden Gate Level I & Level II plasmids (LI & LII)** | |
| --- | --- |
| **Name Description** | |
| *pENTR-pSEC13:SEC13* | Phusion PCR product consisting of *SEC13* genomic construct amplified from *A. thaliana* gDNA with sec13co_FW and sec13co_RV, cloned into *pENTR/D-TOPO* (Invitrogen) via TOPO reaction. |
| LI A-C *pPOLLUX* (GG) | LI promoter element of *POLLUX* (1523 bp). Assembled from 2 PCR fragments amplified from *A. thaliana* gDNA by BpiI cut ligation into LI-BpiI vector.  Fragment 1: AtPol-Pro1+ & AtPol-Pro2-  Fragment 2: AtPol-Pro3+ & AtPol-Pro4- |
| LI C-D *POLLUX* (GG) | LI element containing genomic *POLLUX*. Assembled from 2 PCR fragments amplified from *A. thaliana* gDNA by BpiI cut ligation into LI-BpiI vector.  Fragment 1: AtPol1+ & AtPol2-  Fragment 2: AtPol3+ & AtPol4- |
| LII F 1-2 *pPOLLUX:POLLUX* (GG) | Assembled by BsaI cut ligation from:  LI A-C *pPOLLUX* + LI C-D *POLLUX* + LI dy D-E (BB8) + LI E-F *35S-T* (G59) + LI F-G neo (G3) + LIIc F1-2 (BB30) |
| LII R 3-4 *p35S:mCherry* (GG) | Assembled by BsaI cut ligation from:  LI A-B *p35S* (G5) + LI dy B-C (BB6) + LI C-D *mCherry* (G23) + LI dy D-E (BB8) + LI E-F *nos-T* (G6) + LI dy F-G (G9) + LIIc R 3-4 (BB33) |
| LII R 5-6 *p35S:mCherry* (GG) | Assembled by BsaI cut ligation from:  LI A-B *p35S* (G5) + LI dy B-C (BB6) + LI C-D *mCherry* (G23) + LI dy D-E (BB8) + LI E-F *HSP-T* (G45) + LI dy F-G (G9) + LIIc R 5-6 (BB37) |
| LI A-C *ShRK1* (GG) | Full length genomic DNA from ATG to codon prior to STOP (bases 1-4360); silent mutations introduced to remove type IIS restriction sites; obtained by gene synthesis (GenScript) |
| LI A-C *ShRK2* (GG) | Full length genomic DNA from ATG to codon prior to STOP (bases 1-4185); silent mutations introduced to remove type IIS restriction sites; obtained by gene synthesis (GenScript) |
| LII F 1-2  *pUBi: ShRK1-YFP* (GG) | Assembled by BsaI cut ligation from:  LI A-B *pUBi* (G7) + LI dy B-C (G4) + LI A-C *ShRK1* + LI D-E *YFP* (G12) + LI E-F *35S-T* (G59) + LI F-G hygro (G94) + LIIc F 1-2 (BB30) |
| LII F 1-2  *pUBi: ShRK2-YFP* (GG) | Assembled by BsaI cut ligation from:  LI A-B *pUBi* (G7) + LI dy B-C (G4) + LI A-C *ShRK2* + LI D-E *YFP* (G12) + LI E-F *35S-T* (G59) + LI F-G hygro (G94) + LIIc F 1-2 (BB30) |
| **Plasmids for stable transformation of *A. thaliana*** | |
| **Name** | **Description** |
| *pSEC13:SEC13* + free mCherry | LR reaction of *pENTR-pSEC13:SEC13* and pMDC99 (Curtis & Grossniklaus, 2003); hygromycin resistance |
| *pPOLLUX:POLLUX* + free mCherry (GG) | Assembled by BpiI cut ligation from:  LII F 1-2 *pPOLLUX:POLLUX* + LII 2-3 ins (BB43) + LII R 3-4 *p35S:mCherry* + LII dy 4-6 (BB41) + LIIIα fin (BB45); kanamycin resistance |
| *pUBi:ShRK1-YFP* + free mCherry (GG) | Assembled by BpiI cut ligation from:  LII F 1-2 *pUBi:ShRK1* + LII 2-3 ins (BB43) + LII R 3-4 *p35S:mCherry* + LII dy 4-6 (BB41) + LIIIα fin (BB45); hygromycin resistance |
| *pUBi:ShRK2-YFP* + free mCherry (GG) | Assembled by BpiI cut ligation from:  LII F 1-2 *pUBi:ShRK2* + LII 2-3 ins (BB43) + LII R 3-4 *p35S:mCherry* + LII dy 4-6 (BB41) + LIIIα fin (BB45); hygromycin resistance |
| *pUbi:SYMRK-mOrange (GG)* | (Antolín-Llovera *et al.*, 2014) |
