# Supplementary material for: A set of Arabidopsis genes involved in the accommodation of the downy mildew pathogen Hyaloperonospora arabidopsidis
Source: PLoS Pathog. 2019 Jul 12;15(7):e1007747. doi: 10.1371/journal.ppat.1007747 (PMC6625732; doi:10.1371/journal.ppat.1007747)
Supplement: S3 Table — (DOCX) [file ppat.1007747.s013.docx]

**S3** **Table Oligonucleotides.**

| **Primer sequences for qPCR-based reverse transcript quantification were taken from these publications** | | | |
| --- | --- | --- | --- |
| **Target** | **Reference** | | |
| *PP2A* | (Czechowski, 2005) | | |
| *TIP41-like* | (Czechowski, 2005) | | |
| *FRK1* | (Asai *et al.*, 2002) | | |
| *PDF1.2a* | (Moffat *et al.*, 2012) | | |
| *ERF1* | (Solano *et al.*, 1998) | | |
| *PR1* | (Onate-Sanchez *et al.*, 2006) | | |
| **Plasmid construction** | | |  |
| **Name** | | **Primer sequence (5’ - 3’)** | |
| sec13co_FW  sec13co_RV | | CACCGGGAACACGGGAGAATAG  TTTTGCAATCTCTGTTGTCTGA | |
| N133-pro2_FW  N133-pro3_RV | | AGGGTCTCACACCGTTTTGAAAGACGGCATATTATGG  AGGGTCTCATACAAGGTCTTTTATTGCTTAAAACTCT | |
| N133-pro3_FW  N133_Pro4_RV | | AGGGTCTCATGTACATTTATTTGTTTTCATTGATTG  AGGGTCTCAACATTTTAAACCAGGAAGAGAGCGA | |
| N133_ATG_FW  N133_mut_1b_RW | | AGGGTCTCAATGTTCTCTCCATTGACGAAGA  AGGGTCTCATTTCTTTATCCATTCCACCGGA | |
| N133_mut_1_FW  N133_mut_2_RW | | GGGGTCTCAGAAACCTGTCTTTCTTGGTTTATT  GGGGTCTCAGCGACCGAGAAGCCCT | |
| N133_mut_2_FW  N133_e3_RV | | GGGGTCTCATCGCGTAGTCCTGTTGGTGT  AGGGTCTCACTCTCTGCAGTTGAGTTCCTAGTG | |
| N133_e3_FW  N133_3'UTR2_RV | | AGGGTCTCAAGAGCCTGCGAACTCTCAAA  AGGGTCTCACCTTGGTAGATTCGATACATCATAAAGAGG | |
| AtPol-Pro1+  AtPol-Pro2- | | ATGAAGACTTTACGGGTCTCAGCGGAGCCCAATGACTTCCCACAC  TAGAAGACAAATGACTACAGTTTCATGCCACCA | |
| AtPol-Pro3+  AtPol-Pro4- | | ATGAAGACTTTCATCATTATGCTCATCTTGAATATGT  TAGAAGACAACAGAGGTCTCAGGTGCGGGTTGAAGTAAGTAAATTGAGA | |
| AtPol1+  AtPol2- | | ATGAAGACTTTACGGGTCTCACACCATGCCGATTCATACCCCTAGA  TAGAAGACAACATCTTCTTTCTTCTGATTTGTTCGT | |
| AtPol3+  AtPol4- | | ATGAAGACTTGATGTTCCTTTGAAGAAGAGACTAGC  ATGAAGACTTCAGAGGTCTCACCTTCTGACTTGAGGCGATGACAAC | |
