# Supplementary material for: A set of Arabidopsis genes involved in the accommodation of the downy mildew pathogen Hyaloperonospora arabidopsidis
Source: PLoS Pathog. 2019 Jul 12;15(7):e1007747. doi: 10.1371/journal.ppat.1007747 (PMC6625732; doi:10.1371/journal.ppat.1007747)
Supplement: S4 Table — (DOCX) [file ppat.1007747.s014.docx]

**S4** **Table Mutant lines of *A. thaliana* SNUPO genes and seeds used in the study.**

| **Gene and ID** | **Mutant line** | **Seedbag** |
| --- | --- | --- |
| *ShRK1* (At1g67720) | GK-699C04 2 | 1898 |
| *ShRK2* (At2g37050) | SALK_143700 | 1902 |
| *ShRK1* (At1g67720) x *ShRK2* (At2g37050) | GK-699C04 2 x SALK_143700 | 2118 |
| *Sec13* (At3g01340) x *Nup133* (At2g05120) | SALK_045825C x SALK_092608C | 2130 |
| *Nup133* (At2g05120) | SALK_092608C | 1941 |
| *Pollux* (At5g49960) | SALK_066135C | 2112 |
| *ShRK1* co (At1g67720) | GK-699C04 2 complemented with *pUBi:ShRK1-YFP* + free mCherry | 2089 |
| *ShRK2* co (At2g37050) | SALK_143700 complemented with *pUBi:ShRK2-YFP* + free mCherry | 2079 |
| *Sec13* co (At3g01340) | SALK_045825C complemented with *pSEC13:SEC13* + free mCherry | 2026 |
| *Pollux* co (At5g49960) | SALK_066135C complemented with *pPOLLUX:POLLUX* + free mCherry | 2149 |
